# (*S*)-4,5-Diphenyl-1-[1-phenyl-3-(phenyl­sulfan­yl)propan-2-yl]-2-(thio­phen-2-yl)-1*H*-imidazole

**DOI:** 10.1107/S1600536813032066

**Published:** 2013-11-30

**Authors:** Jie Gao, Hongyan Wang, Liangru Yang, Yongmei Xiao, Pu Mao

**Affiliations:** a School of Chemistry and Chemical Engineering, Henan University of Technology, Zhengzhou 450001, People’s Republic of China

## Abstract

In the title compound, C_34_H_28_N_2_S_2_, the central imidazole ring (r.m.s. deviation = 0.0015 Å) forms dihedral angles of 55.7 (3), 17.94 (11) and 86.27 (11)°, respectively, with the mean planes of the attached thienyl and two phenyl substituents. The thienyl ring shows ring-flip disorder [occupancy ratio = 0.647 (2):0.353 (2)]. The chiral centre maintains the *S* configuration of the l-phenyl­alaninol starting material. Intra- and inter­molecular C—H⋯S hydrogen bonds involving the disordered thienyl ring are observed.

## Related literature

For the synthesis of aryl sulfides, see: Mispelaere-Canivet *et al.* (2005 ▶); Zhang *et al.* (2007 ▶); Wu *et al.* (2009 ▶); Lv & Bao (2007 ▶). For related compounds synthesized by our group, see: Mao *et al.* (2010 ▶); Yang *et al.* (2012 ▶); Xiao *et al.* (2012 ▶); Gao *et al.* (2013 ▶).
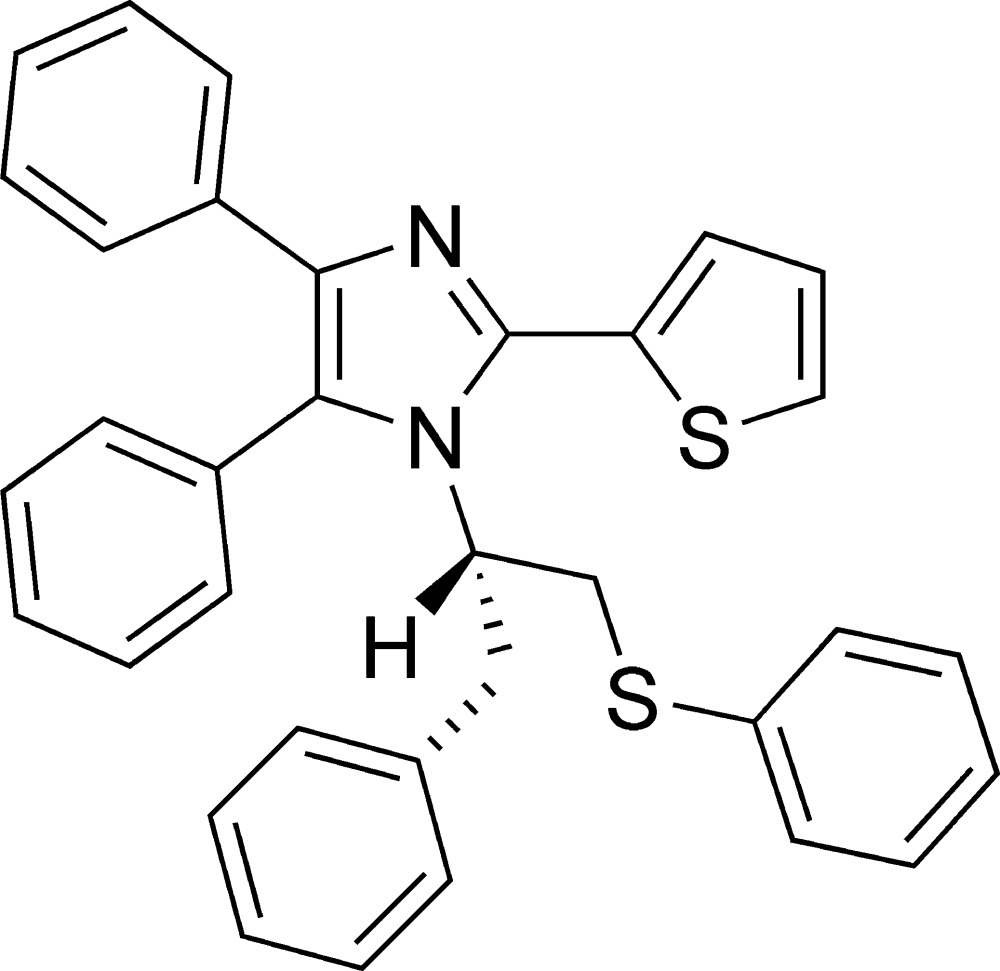


## Experimental

### 

#### Crystal data

C_34_H_28_N_2_S_2_
*M* *_r_* = 528.70
Orthorhombic, 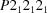

*a* = 12.7882 (7) Å
*b* = 13.7906 (6) Å
*c* = 16.0636 (7) Å
*V* = 2832.9 (2) Å^3^
*Z* = 4
Cu *K*α radiation
μ = 1.89 mm^−1^
*T* = 291 K
0.26 × 0.23 × 0.2 mm

#### Data collection

Oxford Diffraction Xcalibur (Eos, Gemini) diffractometer
Absorption correction: multi-scan *CrysAlis PRO*; Agilent, 2011 ▶ *T*_min_ = 0.906, *T*_max_ = 1.000
10599 measured reflections
5065 independent reflections
4456 reflections with *I* > 2σ(*I*)
*R*_int_ = 0.021

#### Refinement

*R*[*F* ^2^ > 2σ(*F* ^2^)] = 0.045
*wR*(*F* ^2^) = 0.127
*S* = 1.03
5065 reflections
356 parameters
18 restraints
H-atom parameters constrained
Δρ_max_ = 0.19 e Å^−3^
Δρ_min_ = −0.17 e Å^−3^
Absolute structure: Flack (1983 ▶), 2202 Friedel pairs
Absolute structure parameter: 0.00 (2)

### 

Data collection: *CrysAlis PRO* (Agilent, 2011 ▶); cell refinement: *CrysAlis PRO*; data reduction: *CrysAlis PRO*; program(s) used to solve structure: *SHELXS97* (Sheldrick, 2008 ▶); program(s) used to refine structure: *SHELXL97* (Sheldrick, 2008 ▶); molecular graphics: *OLEX2* (Dolomanov *et al.*, 2009 ▶); software used to prepare material for publication: *OLEX2*.

## Supplementary Material

Crystal structure: contains datablock(s) I, Global. DOI: 10.1107/S1600536813032066/rz5094sup1.cif

Structure factors: contains datablock(s) I. DOI: 10.1107/S1600536813032066/rz5094Isup2.hkl

Supplementary material file. DOI: 10.1107/S1600536813032066/rz5094Isup3.cml

Additional supplementary materials:  crystallographic information; 3D view; checkCIF report

## Figures and Tables

**Table 1:** Hydrogen-bond geometry (Å, °)

*D*—H⋯*A*	*D*—H	H⋯*A*	*D*⋯*A*	*D*—H⋯*A*
C21—H21*B*⋯S1^i^	0.97	2.75	3.677 (3)	161
C22—H22⋯S1*A*	0.98	2.73	3.468 (5)	132

Symmetry code: (i) 
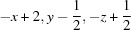
.

**Fig. 1.**
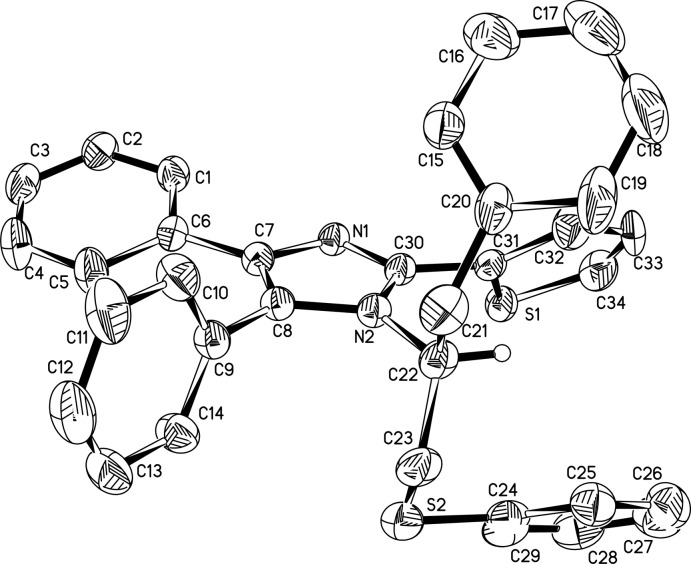
The molecular structure of the title compound showing 30% probability displacement ellipsoids. Hydrogen atoms, except for that associated to the chiral C22 atom, are omitted for clarity.

## supplementary crystallographic information

### 1. Comment

Aryl sulfides are important organic compounds with biological, pharmaceutical, and materials interest. Among various methods for the preparation of S-containing compounds, transitionmetal-catalyzed C—S formation has become the most versatile strategy in modern organic chemistry (Mispelaere-Canivet *et al.*, 2005; Zhang *et al.*, 2007; Wu *et al.*, 2009; Lv *et al.*, 2007). Our group is interested in the synthesis and application of chiral imidazolium derived from natural amino acids (Mao *et al.*, 2010; Yang *et al.*, 2012; Xiao *et al.*, 2012; Gao *et al.*, 2013). During the study, we observed that the condensation of *L*-phenylalaninol, dibenzoyl, thiophene-2-carbaldehyde and ammonium acetate afforded (*S*)-2-(4,5-diphenyl-2-(thiophen-2-yl)-1*H*-imidazol-1-yl)-3-phenylpropan-1-ol (I), which was converted to *p*-toluenesulfonate (II) upon treatment with *p*-toluensulfonyl chloride. The following reaction of II with thiophenol catalyzed by CuI under basic condition produced the title compound (III) smoothly.

The molecular structure of the title compound (III) is shown in Figure 1. The imidazole ring (C7/C8/N2/C30/N1) is essentially planar, the maximum deviation being 0.002 (2) Å for atom C7. The thienyl ring shows ring-flip disorder, the major and minor components of the disorder having an occupancy factor of 0.647 (2) and 0.353 (2), respectively. The dihedral angle between the mean plane through the thienyl ring and the imidazole ring is 55.7 (3)°. The dihedral angles between the two phenyl substituents (C1—C6, C9—C14) and the imidazole ring are 17.94 (11) and 86.27 (11)°. The chiral C22 carbon atom maintains the *S* configuration of the *L*-phenylalaninol starting material. In the crystal structure, intra- and intermolecular C—H···S hydrogen bonds involving the disordered thienyl ring (Table 1) are observed.

### 2. Experimental

NaH (0.048 g, 2 mmol) was added to an anhydrous 1,4-dioxane (20 ml) solution containing compound I (0.087 g, 0.2 mmol) and the mixture was kept at r.t. for 0.5 h. *p*-Toluenesulfonyl chloride (0.114 g, 0.6 mmol) was then added and the reaction was followed by TLC detection until the raw material disappeared. Evaporation of the solvent gave the crude product of *p*-toluenesulfonate (II), which was then purified by silica column chromatography. In a 50 ml flask, *p*-toluenesulfonate (II, 0.295 g, 0.5 mmol), thiophenol (0.110 g, 1 mmol), cuprous iodide (0.001 g, 0.005 mmol), and potassium hydroxide (0.056 g, 1 mmol) were dissolved in 1,4-dioxane (10 ml), and the solution was heated to 120°C for 12 h under an argon atmosphere. The volatile compounds were then removed *in vacuo* and a brown, oily residue remained, which was purified by silica column chromatography. Crystallization in MeOH afforded colourless crystals of the title compound (III).

### 3. Refinement

The S–C and C—C bond distances involving the disordered S1, S1A, C32, C32A, C33, C33A and C34A atoms were constrained to be 2.5 (2) and 1.4 (2) Å, respectively. The ADPs of atom C17 were restrained to be nearly isotropic. H atoms were placed geometrically and refined as riding, with C—H = 0.93–0.98 Å, and with *U*_iso_(H) = 1.2 *U*_eq_(C).

### Crystal data

**Table:**

C_34_H_28_N_2_S_2_	*D*_x_ = 1.240 Mg m^−^^3^
*M**_r_* = 528.70	Cu *K*α radiation, λ = 1.5418 Å
Orthorhombic, *P*2_1_2_1_2_1_	Cell parameters from 4362 reflections
*a* = 12.7882 (7) Å	θ = 5.4–72.0°
*b* = 13.7906 (6) Å	µ = 1.89 mm^−^^1^
*c* = 16.0636 (7) Å	*T* = 291 K
*V* = 2832.9 (2) Å^3^	Prism, colourless
*Z* = 4	0.26 × 0.23 × 0.2 mm
*F*(000) = 1112	

### Data collection

**Table:**

Oxford Diffraction Xcalibur (Eos, Gemini) diffractometer	5065 independent reflections
Radiation source: Enhance (Cu) X-ray Source	4456 reflections with *I* > 2σ(*I*)
Graphite monochromator	*R*_int_ = 0.021
Detector resolution: 16.2312 pixels mm^-1^	θ_max_ = 67.1°, θ_min_ = 4.2°
ω scans	*h* = −15→14
Absorption correction: multi-scan *CrysAlis PRO*; Agilent, 2011	*k* = −16→16
*T*_min_ = 0.906, *T*_max_ = 1.000	*l* = −19→13
10599 measured reflections	

### Refinement

**Table:**

Refinement on *F*^2^	Secondary atom site location: difference Fourier map
Least-squares matrix: full	Hydrogen site location: inferred from neighbouring sites
*R*[*F*^2^ > 2σ(*F*^2^)] = 0.045	H-atom parameters constrained
*wR*(*F*^2^) = 0.127	*w* = 1/[σ^2^(*F*_o_^2^) + (0.0605*P*)^2^ + 0.3647*P*] where *P* = (*F*_o_^2^ + 2*F*_c_^2^)/3
*S* = 1.03	(Δ/σ)_max_ < 0.001
5065 reflections	Δρ_max_ = 0.19 e Å^−^^3^
356 parameters	Δρ_min_ = −0.17 e Å^−^^3^
18 restraints	Absolute structure: Flack (1983), 2202 Friedel pairs
Primary atom site location: structure-invariant direct methods	Absolute structure parameter: 0.00 (2)

### Special details

**Table:**

Geometry. All e.s.d.'s (except the e.s.d. in the dihedral angle between two l.s. planes) are estimated using the full covariance matrix. The cell e.s.d.'s are taken into account individually in the estimation of e.s.d.'s in distances, angles and torsion angles; correlations between e.s.d.'s in cell parameters are only used when they are defined by crystal symmetry. An approximate (isotropic) treatment of cell e.s.d.'s is used for estimating e.s.d.'s involving l.s. planes.
Refinement. Refinement of *F*^2^ against ALL reflections. The weighted *R*-factor *wR* and goodness of fit *S* are based on *F*^2^, conventional *R*-factors *R* are based on *F*, with *F* set to zero for negative *F*^2^. The threshold expression of *F*^2^ > σ(*F*^2^) is used only for calculating *R*-factors(gt) *etc*. and is not relevant to the choice of reflections for refinement. *R*-factors based on *F*^2^ are statistically about twice as large as those based on *F*, and *R*- factors based on ALL data will be even larger.

### Fractional atomic coordinates and isotropic or equivalent isotropic displacement parameters (Å^2^)

**Table:**

	*x*	*y*	*z*	*U*_iso_*/*U*_eq_	Occ. (<1)
S1	0.81367 (14)	0.86015 (13)	0.18676 (10)	0.0729 (4)	0.647 (2)
S1A	1.0259 (4)	0.8013 (3)	0.1389 (3)	0.0729 (4)	0.353 (2)
S2	0.81179 (9)	0.59192 (7)	0.34985 (7)	0.1012 (3)	
N1	0.94171 (17)	0.89681 (13)	0.35239 (13)	0.0577 (5)	
N2	0.99498 (16)	0.74395 (13)	0.34692 (13)	0.0580 (5)	
C1	0.9468 (3)	1.0322 (2)	0.48649 (18)	0.0746 (7)	
H1	0.9486	1.0543	0.4318	0.089*	
C2	0.9363 (3)	1.0980 (2)	0.5507 (2)	0.0894 (10)	
H2	0.9311	1.1639	0.5388	0.107*	
C3	0.9334 (3)	1.0675 (3)	0.6313 (2)	0.0899 (10)	
H3	0.9279	1.1123	0.6743	0.108*	
C4	0.9387 (4)	0.9715 (3)	0.6481 (2)	0.1072 (13)	
H4	0.9358	0.9504	0.7030	0.129*	
C5	0.9485 (4)	0.9043 (3)	0.5844 (2)	0.0957 (11)	
H5	0.9510	0.8384	0.5969	0.115*	
C6	0.9547 (2)	0.9346 (2)	0.50214 (16)	0.0636 (6)	
C7	0.9663 (2)	0.86640 (18)	0.43152 (15)	0.0586 (5)	
C8	0.9998 (2)	0.77216 (18)	0.43000 (16)	0.0581 (5)	
C9	1.0384 (2)	0.70951 (18)	0.49927 (17)	0.0636 (6)	
C10	1.1412 (3)	0.7148 (3)	0.5238 (2)	0.0880 (10)	
H10	1.1864	0.7573	0.4969	0.106*	
C11	1.1781 (3)	0.6576 (4)	0.5881 (2)	0.1129 (15)	
H11	1.2481	0.6608	0.6035	0.136*	
C12	1.1111 (5)	0.5961 (3)	0.6291 (3)	0.1145 (15)	
H12	1.1359	0.5573	0.6722	0.137*	
C13	1.0084 (4)	0.5919 (3)	0.6068 (3)	0.1089 (14)	
H13	0.9630	0.5510	0.6352	0.131*	
C14	0.9716 (3)	0.6484 (2)	0.5419 (2)	0.0862 (9)	
H14	0.9014	0.6452	0.5270	0.103*	
C15	1.2350 (3)	0.7748 (3)	0.2846 (2)	0.0915 (10)	
H15	1.2166	0.7990	0.3366	0.110*	
C16	1.2929 (4)	0.8319 (5)	0.2318 (3)	0.1296 (17)	
H16	1.3138	0.8938	0.2476	0.156*	
C17	1.3196 (5)	0.7947 (6)	0.1542 (4)	0.162 (2)	
H17	1.3566	0.8323	0.1162	0.195*	
C18	1.2909 (5)	0.7026 (6)	0.1346 (4)	0.171 (3)	
H18	1.3122	0.6768	0.0839	0.205*	
C19	1.2322 (4)	0.6470 (5)	0.1861 (3)	0.140 (2)	
H19	1.2117	0.5852	0.1698	0.169*	
C20	1.2032 (2)	0.6824 (3)	0.2626 (2)	0.0829 (9)	
C21	1.1339 (3)	0.6223 (2)	0.3168 (3)	0.0912 (10)	
H21A	1.1560	0.6298	0.3742	0.109*	
H21B	1.1426	0.5546	0.3019	0.109*	
C22	1.0172 (2)	0.64862 (18)	0.3103 (2)	0.0718 (7)	
H22	1.0012	0.6537	0.2508	0.086*	
C23	0.9491 (3)	0.5675 (2)	0.3445 (2)	0.0892 (9)	
H23A	0.9734	0.5518	0.4001	0.107*	
H23B	0.9594	0.5105	0.3101	0.107*	
C24	0.7696 (3)	0.5971 (2)	0.2451 (3)	0.0890 (9)	
C25	0.8234 (3)	0.5596 (3)	0.1775 (3)	0.1055 (11)	
H25	0.8884	0.5308	0.1856	0.127*	
C26	0.7819 (4)	0.5644 (4)	0.0983 (3)	0.1259 (17)	
H26	0.8187	0.5382	0.0538	0.151*	
C27	0.6879 (4)	0.6071 (4)	0.0850 (4)	0.1328 (17)	
H27	0.6607	0.6113	0.0314	0.159*	
C28	0.6332 (4)	0.6443 (4)	0.1515 (4)	0.1305 (17)	
H28	0.5681	0.6727	0.1429	0.157*	
C29	0.6739 (3)	0.6397 (3)	0.2305 (3)	0.1080 (13)	
H29	0.6364	0.6658	0.2748	0.130*	
C30	0.9593 (2)	0.82233 (16)	0.30391 (15)	0.0548 (5)	
C31	0.9363 (2)	0.82435 (16)	0.21422 (16)	0.0590 (6)	
C32	0.9968 (7)	0.8110 (9)	0.1418 (7)	0.108 (4)	0.647 (2)
H32	1.0664	0.7912	0.1424	0.130*	0.647 (2)
C32A	0.8446 (13)	0.8515 (15)	0.1757 (10)	0.108 (4)	0.353 (2)
H32A	0.7844	0.8649	0.2060	0.130*	0.353 (2)
C33	0.9421 (13)	0.8304 (11)	0.0703 (5)	0.099 (3)	0.647 (2)
H33	0.9687	0.8242	0.0167	0.118*	0.647 (2)
C33A	0.847 (3)	0.858 (3)	0.0864 (10)	0.099 (3)	0.353 (2)
H33A	0.7910	0.8752	0.0520	0.118*	0.353 (2)
C34	0.8463 (12)	0.8591 (13)	0.0882 (7)	0.094 (3)	0.647 (2)
H34	0.7996	0.8778	0.0469	0.113*	0.647 (2)
C34A	0.946 (2)	0.835 (2)	0.0614 (14)	0.094 (3)	0.353 (2)
H34A	0.9678	0.8376	0.0061	0.113*	0.353 (2)

### Atomic displacement parameters (Å^2^)

**Table:**

	*U*^11^	*U*^22^	*U*^33^	*U*^12^	*U*^13^	*U*^23^
S1	0.0778 (9)	0.0781 (7)	0.0629 (6)	0.0021 (6)	−0.0096 (5)	0.0013 (5)
S1A	0.0778 (9)	0.0781 (7)	0.0629 (6)	0.0021 (6)	−0.0096 (5)	0.0013 (5)
S2	0.1050 (6)	0.0971 (6)	0.1016 (6)	−0.0232 (5)	0.0174 (5)	−0.0074 (5)
N1	0.0672 (12)	0.0472 (9)	0.0586 (11)	0.0048 (9)	−0.0024 (10)	−0.0020 (8)
N2	0.0631 (11)	0.0494 (9)	0.0614 (11)	0.0049 (8)	−0.0007 (9)	0.0001 (9)
C1	0.092 (2)	0.0657 (15)	0.0662 (16)	0.0033 (15)	0.0043 (15)	−0.0062 (12)
C2	0.111 (3)	0.0676 (16)	0.090 (2)	0.0007 (18)	0.0077 (19)	−0.0194 (16)
C3	0.095 (2)	0.093 (2)	0.081 (2)	0.0054 (18)	0.0012 (18)	−0.0309 (17)
C4	0.143 (4)	0.122 (3)	0.0566 (17)	0.029 (3)	0.003 (2)	−0.0110 (19)
C5	0.138 (3)	0.084 (2)	0.0645 (17)	0.020 (2)	0.0056 (19)	−0.0057 (15)
C6	0.0631 (14)	0.0697 (14)	0.0579 (13)	0.0050 (12)	−0.0044 (12)	−0.0070 (11)
C7	0.0614 (13)	0.0564 (12)	0.0581 (13)	0.0033 (11)	−0.0056 (10)	0.0021 (10)
C8	0.0581 (13)	0.0563 (12)	0.0599 (13)	0.0021 (10)	−0.0029 (11)	0.0039 (10)
C9	0.0706 (15)	0.0593 (12)	0.0610 (13)	0.0089 (12)	−0.0025 (12)	0.0053 (11)
C10	0.0692 (18)	0.118 (3)	0.0768 (19)	0.0072 (18)	−0.0058 (15)	0.0165 (18)
C11	0.093 (3)	0.166 (4)	0.079 (2)	0.047 (3)	−0.017 (2)	0.013 (3)
C12	0.160 (4)	0.102 (3)	0.081 (2)	0.044 (3)	−0.015 (3)	0.023 (2)
C13	0.154 (4)	0.088 (2)	0.085 (2)	−0.009 (3)	0.003 (2)	0.0303 (19)
C14	0.089 (2)	0.0846 (19)	0.085 (2)	−0.0069 (17)	−0.0022 (17)	0.0202 (17)
C15	0.091 (2)	0.103 (2)	0.080 (2)	0.0019 (19)	−0.0002 (17)	−0.0148 (19)
C16	0.088 (3)	0.172 (5)	0.129 (4)	−0.022 (3)	0.003 (3)	0.021 (4)
C17	0.109 (3)	0.236 (6)	0.143 (4)	−0.015 (4)	0.045 (3)	0.031 (4)
C18	0.142 (4)	0.264 (6)	0.107 (3)	0.017 (5)	0.051 (3)	−0.032 (4)
C19	0.096 (3)	0.216 (6)	0.110 (3)	0.022 (3)	0.008 (2)	−0.085 (4)
C20	0.0703 (18)	0.102 (2)	0.0767 (18)	0.0235 (16)	−0.0040 (14)	−0.0269 (17)
C21	0.092 (2)	0.0697 (18)	0.112 (3)	0.0286 (16)	−0.003 (2)	−0.0105 (18)
C22	0.0861 (19)	0.0494 (12)	0.0799 (17)	0.0094 (12)	−0.0020 (14)	−0.0054 (12)
C23	0.114 (3)	0.0509 (13)	0.103 (2)	−0.0030 (15)	−0.006 (2)	0.0003 (15)
C24	0.080 (2)	0.0751 (18)	0.111 (3)	−0.0192 (16)	0.0114 (19)	−0.0018 (18)
C25	0.082 (2)	0.122 (3)	0.112 (3)	−0.007 (2)	0.008 (2)	−0.002 (2)
C26	0.109 (3)	0.170 (5)	0.099 (3)	−0.032 (3)	0.005 (3)	−0.002 (3)
C27	0.107 (3)	0.159 (5)	0.132 (4)	−0.035 (4)	−0.015 (3)	0.024 (4)
C28	0.100 (3)	0.120 (4)	0.171 (5)	−0.012 (3)	−0.025 (4)	0.008 (4)
C29	0.094 (3)	0.084 (2)	0.146 (4)	−0.012 (2)	0.004 (3)	−0.008 (2)
C30	0.0603 (13)	0.0486 (11)	0.0556 (12)	0.0022 (10)	−0.0015 (10)	−0.0009 (9)
C31	0.0662 (15)	0.0494 (11)	0.0615 (13)	−0.0035 (11)	−0.0043 (11)	−0.0035 (10)
C32	0.097 (7)	0.131 (7)	0.096 (5)	0.002 (5)	−0.012 (5)	−0.023 (4)
C32A	0.097 (7)	0.131 (7)	0.096 (5)	0.002 (5)	−0.012 (5)	−0.023 (4)
C33	0.125 (7)	0.127 (7)	0.044 (3)	−0.004 (5)	0.015 (3)	−0.024 (4)
C33A	0.125 (7)	0.127 (7)	0.044 (3)	−0.004 (5)	0.015 (3)	−0.024 (4)
C34	0.087 (5)	0.112 (6)	0.084 (4)	−0.001 (4)	−0.033 (4)	0.003 (4)
C34A	0.087 (5)	0.112 (6)	0.084 (4)	−0.001 (4)	−0.033 (4)	0.003 (4)

### Geometric parameters (Å, º)

**Table:**

S1—C31	1.702 (3)	C16—C17	1.391 (8)
S1—C34	1.637 (12)	C17—H17	0.9300
S1A—C31	1.696 (5)	C17—C18	1.359 (9)
S1A—C34A	1.673 (18)	C18—H18	0.9300
S2—C23	1.789 (4)	C18—C19	1.355 (9)
S2—C24	1.769 (4)	C19—H19	0.9300
N1—C7	1.375 (3)	C19—C20	1.373 (5)
N1—C30	1.308 (3)	C20—C21	1.493 (5)
N2—C8	1.392 (3)	C21—H21A	0.9700
N2—C22	1.468 (3)	C21—H21B	0.9700
N2—C30	1.361 (3)	C21—C22	1.539 (4)
C1—H1	0.9300	C22—H22	0.9800
C1—C2	1.381 (4)	C22—C23	1.521 (4)
C1—C6	1.373 (4)	C23—H23A	0.9700
C2—H2	0.9300	C23—H23B	0.9700
C2—C3	1.361 (5)	C24—C25	1.386 (5)
C3—H3	0.9300	C24—C29	1.377 (6)
C3—C4	1.353 (5)	C25—H25	0.9300
C4—H4	0.9300	C25—C26	1.379 (6)
C4—C5	1.386 (5)	C26—H26	0.9300
C5—H5	0.9300	C26—C27	1.355 (8)
C5—C6	1.388 (4)	C27—H27	0.9300
C6—C7	1.481 (3)	C27—C28	1.376 (7)
C7—C8	1.369 (4)	C28—H28	0.9300
C8—C9	1.493 (3)	C28—C29	1.374 (7)
C9—C10	1.374 (4)	C29—H29	0.9300
C9—C14	1.382 (4)	C30—C31	1.471 (3)
C10—H10	0.9300	C31—C32	1.410 (11)
C10—C11	1.383 (5)	C31—C32A	1.377 (16)
C11—H11	0.9300	C32—H32	0.9300
C11—C12	1.373 (6)	C32—C33	1.371 (11)
C12—H12	0.9300	C32A—H32A	0.9300
C12—C13	1.363 (7)	C32A—C33A	1.438 (18)
C13—H13	0.9300	C33—H33	0.9300
C13—C14	1.383 (5)	C33—C34	1.318 (12)
C14—H14	0.9300	C33A—H33A	0.9300
C15—H15	0.9300	C33A—C34A	1.375 (18)
C15—C16	1.374 (6)	C34—H34	0.9300
C15—C20	1.383 (5)	C34A—H34A	0.9300
C16—H16	0.9300		
			
C34—S1—C31	90.7 (5)	C19—C20—C21	119.0 (4)
C34A—S1A—C31	93.9 (10)	C20—C21—H21A	108.8
C24—S2—C23	105.15 (18)	C20—C21—H21B	108.8
C30—N1—C7	105.8 (2)	C20—C21—C22	113.9 (3)
C8—N2—C22	128.8 (2)	H21A—C21—H21B	107.7
C30—N2—C8	106.22 (19)	C22—C21—H21A	108.8
C30—N2—C22	124.9 (2)	C22—C21—H21B	108.8
C2—C1—H1	119.5	N2—C22—C21	111.8 (2)
C6—C1—H1	119.5	N2—C22—H22	106.6
C6—C1—C2	121.0 (3)	N2—C22—C23	113.7 (3)
C1—C2—H2	119.7	C21—C22—H22	106.6
C3—C2—C1	120.6 (3)	C23—C22—C21	111.0 (3)
C3—C2—H2	119.7	C23—C22—H22	106.6
C2—C3—H3	120.3	S2—C23—H23A	108.2
C4—C3—C2	119.4 (3)	S2—C23—H23B	108.2
C4—C3—H3	120.3	C22—C23—S2	116.2 (2)
C3—C4—H4	119.6	C22—C23—H23A	108.2
C3—C4—C5	120.8 (3)	C22—C23—H23B	108.2
C5—C4—H4	119.6	H23A—C23—H23B	107.4
C4—C5—H5	119.8	C25—C24—S2	125.4 (3)
C4—C5—C6	120.4 (3)	C29—C24—S2	116.7 (3)
C6—C5—H5	119.8	C29—C24—C25	117.9 (4)
C1—C6—C5	117.7 (3)	C24—C25—H25	119.6
C1—C6—C7	119.3 (2)	C26—C25—C24	120.9 (4)
C5—C6—C7	122.9 (3)	C26—C25—H25	119.6
N1—C7—C6	119.5 (2)	C25—C26—H26	119.7
C8—C7—N1	110.2 (2)	C27—C26—C25	120.6 (5)
C8—C7—C6	130.4 (2)	C27—C26—H26	119.7
N2—C8—C9	124.6 (2)	C26—C27—H27	120.4
C7—C8—N2	105.6 (2)	C26—C27—C28	119.3 (5)
C7—C8—C9	129.8 (2)	C28—C27—H27	120.4
C10—C9—C8	120.0 (3)	C27—C28—H28	119.7
C10—C9—C14	118.8 (3)	C29—C28—C27	120.5 (5)
C14—C9—C8	121.2 (3)	C29—C28—H28	119.7
C9—C10—H10	119.6	C24—C29—H29	119.6
C9—C10—C11	120.7 (4)	C28—C29—C24	120.9 (5)
C11—C10—H10	119.6	C28—C29—H29	119.6
C10—C11—H11	120.1	N1—C30—N2	112.3 (2)
C12—C11—C10	119.8 (4)	N1—C30—C31	122.2 (2)
C12—C11—H11	120.1	N2—C30—C31	125.4 (2)
C11—C12—H12	120.0	S1A—C31—S1	119.4 (2)
C13—C12—C11	120.1 (3)	C30—C31—S1	116.4 (2)
C13—C12—H12	120.0	C30—C31—S1A	124.1 (2)
C12—C13—H13	119.9	C32—C31—S1	109.3 (4)
C12—C13—C14	120.2 (4)	C32—C31—C30	134.1 (5)
C14—C13—H13	119.9	C32A—C31—S1A	107.8 (7)
C9—C14—C13	120.4 (4)	C32A—C31—C30	128.0 (7)
C9—C14—H14	119.8	C32A—C31—C32	97.6 (7)
C13—C14—H14	119.8	C31—C32—H32	123.7
C16—C15—H15	119.0	C33—C32—C31	112.6 (8)
C16—C15—C20	121.9 (4)	C33—C32—H32	123.7
C20—C15—H15	119.0	C31—C32A—H32A	121.6
C15—C16—H16	120.8	C31—C32A—C33A	116.8 (17)
C15—C16—C17	118.3 (6)	C33A—C32A—H32A	121.6
C17—C16—H16	120.8	C32—C33—H33	124.8
C16—C17—H17	120.5	C34—C33—C32	110.5 (9)
C18—C17—C16	119.1 (6)	C34—C33—H33	124.8
C18—C17—H17	120.5	C32A—C33A—H33A	126.5
C17—C18—H18	118.8	C34A—C33A—C32A	107 (2)
C19—C18—C17	122.5 (6)	C34A—C33A—H33A	126.5
C19—C18—H18	118.8	S1—C34—H34	121.6
C18—C19—H19	120.1	C33—C34—S1	116.8 (8)
C18—C19—C20	119.7 (6)	C33—C34—H34	121.6
C20—C19—H19	120.1	S1A—C34A—H34A	122.9
C15—C20—C21	122.5 (3)	C33A—C34A—S1A	114 (2)
C19—C20—C15	118.4 (4)	C33A—C34A—H34A	122.9
			
S1—C31—C32—C33	0.3 (12)	C15—C20—C21—C22	80.1 (4)
S1—C31—C32A—C33A	177 (6)	C16—C15—C20—C19	1.0 (6)
S1A—C31—C32—C33	176 (4)	C16—C15—C20—C21	−175.5 (4)
S1A—C31—C32A—C33A	−3 (2)	C16—C17—C18—C19	3.6 (12)
S2—C24—C25—C26	177.7 (4)	C17—C18—C19—C20	−2.5 (11)
S2—C24—C29—C28	−177.9 (3)	C18—C19—C20—C15	0.1 (7)
N1—C7—C8—N2	−0.3 (3)	C18—C19—C20—C21	176.8 (5)
N1—C7—C8—C9	177.5 (3)	C19—C20—C21—C22	−96.4 (4)
N1—C30—C31—S1	−51.8 (3)	C20—C15—C16—C17	0.1 (7)
N1—C30—C31—S1A	123.7 (3)	C20—C21—C22—N2	−69.4 (4)
N1—C30—C31—C32	121.4 (7)	C20—C21—C22—C23	162.5 (3)
N1—C30—C31—C32A	−50.8 (12)	C21—C22—C23—S2	174.6 (2)
N2—C8—C9—C10	93.9 (4)	C22—N2—C8—C7	−176.8 (3)
N2—C8—C9—C14	−88.7 (4)	C22—N2—C8—C9	5.3 (4)
N2—C22—C23—S2	47.4 (4)	C22—N2—C30—N1	177.2 (2)
N2—C30—C31—S1	124.4 (2)	C22—N2—C30—C31	0.6 (4)
N2—C30—C31—S1A	−60.1 (4)	C23—S2—C24—C25	18.5 (4)
N2—C30—C31—C32	−62.3 (8)	C23—S2—C24—C29	−163.3 (3)
N2—C30—C31—C32A	125.4 (12)	C24—S2—C23—C22	68.8 (3)
C1—C2—C3—C4	−1.4 (7)	C24—C25—C26—C27	0.8 (8)
C1—C6—C7—N1	−17.1 (4)	C25—C24—C29—C28	0.4 (6)
C1—C6—C7—C8	162.5 (3)	C25—C26—C27—C28	−1.1 (8)
C2—C1—C6—C5	1.9 (5)	C26—C27—C28—C29	1.1 (8)
C2—C1—C6—C7	−179.8 (3)	C27—C28—C29—C24	−0.8 (7)
C2—C3—C4—C5	0.9 (7)	C29—C24—C25—C26	−0.5 (6)
C3—C4—C5—C6	1.0 (7)	C30—N1—C7—C6	−180.0 (2)
C4—C5—C6—C1	−2.4 (6)	C30—N1—C7—C8	0.4 (3)
C4—C5—C6—C7	179.4 (4)	C30—N2—C8—C7	0.1 (3)
C5—C6—C7—N1	161.1 (3)	C30—N2—C8—C9	−177.8 (2)
C5—C6—C7—C8	−19.3 (5)	C30—N2—C22—C21	116.0 (3)
C6—C1—C2—C3	0.0 (6)	C30—N2—C22—C23	−117.3 (3)
C6—C7—C8—N2	−179.9 (3)	C30—C31—C32—C33	−173.3 (8)
C6—C7—C8—C9	−2.1 (5)	C30—C31—C32A—C33A	172.6 (18)
C7—N1—C30—N2	−0.3 (3)	C31—S1—C34—C33	2.5 (15)
C7—N1—C30—C31	176.3 (2)	C31—S1A—C34A—C33A	−5 (3)
C7—C8—C9—C10	−83.5 (4)	C31—C32—C33—C34	1.5 (18)
C7—C8—C9—C14	93.9 (4)	C31—C32A—C33A—C34A	0 (4)
C8—N2—C22—C21	−67.7 (4)	C32—C31—C32A—C33A	−2 (2)
C8—N2—C22—C23	59.0 (4)	C32—C33—C34—S1	−3 (2)
C8—N2—C30—N1	0.2 (3)	C32A—C31—C32—C33	0.5 (12)
C8—N2—C30—C31	−176.4 (2)	C32A—C33A—C34A—S1A	4 (3)
C8—C9—C10—C11	179.7 (3)	C34—S1—C31—S1A	−2.3 (7)
C8—C9—C14—C13	−179.0 (3)	C34—S1—C31—C30	173.4 (7)
C9—C10—C11—C12	−1.3 (6)	C34—S1—C31—C32	−1.4 (9)
C10—C9—C14—C13	−1.6 (5)	C34—S1—C31—C32A	−3 (5)
C10—C11—C12—C13	−0.4 (7)	C34A—S1A—C31—S1	3.8 (11)
C11—C12—C13—C14	1.0 (7)	C34A—S1A—C31—C30	−171.5 (10)
C12—C13—C14—C9	0.0 (6)	C34A—S1A—C31—C32	−1 (4)
C14—C9—C10—C11	2.2 (5)	C34A—S1A—C31—C32A	3.9 (15)
C15—C16—C17—C18	−2.3 (10)		

### Hydrogen-bond geometry (Å, º)

**Table:**

*D*—H···*A*	*D*—H	H···*A*	*D*···*A*	*D*—H···*A*
C21—H21*B*···S1^i^	0.97	2.75	3.677 (3)	161
C22—H22···S1*A*	0.98	2.73	3.468 (5)	132

Symmetry code: (i) −*x*+2, *y*−1/2, −*z*+1/2.
